# Gene expression and pathway analysis of ovarian cancer cells selected for resistance to cisplatin, paclitaxel, or doxorubicin

**DOI:** 10.1186/1757-2215-4-21

**Published:** 2011-12-05

**Authors:** Cheryl A Sherman-Baust, Kevin G Becker, William H Wood III, Yongqing Zhang, Patrice J Morin

**Affiliations:** 1 Laboratory of Molecular Biology and Immunology, National Institute on Aging, Baltimore MD 21224, USA; 2 Research Resource Branch, National Institute on Aging, Baltimore MD 21224, USA; 3 Department of Pathology, Johns Hopkins Medical Institutions, Baltimore, MD 21287, USA

## Abstract

**Background:**

Resistance to current chemotherapeutic agents is a major cause of therapy failure in ovarian cancer patients, but the exact mechanisms leading to the development of drug resistance remain unclear.

**Methods:**

To better understand mechanisms of drug resistance, and possibly identify novel targets for therapy, we generated a series of drug resistant ovarian cancer cell lines through repeated exposure to three chemotherapeutic drugs (cisplatin, doxorubicin, or paclitaxel), and identified changes in gene expression patterns using Illumina whole-genome expression microarrays. Validation of selected genes was performed by RT-PCR and immunoblotting. Pathway enrichment analysis using the KEGG, GO, and Reactome databases was performed to identify pathways that may be important in each drug resistance phenotype.

**Results:**

A total of 845 genes (p < 0.01) were found altered in at least one drug resistance phenotype when compared to the parental, drug sensitive cell line. Focusing on each resistance phenotype individually, we identified 460, 366, and 337 genes significantly altered in cells resistant to cisplatin, doxorubicin, and paclitaxel, respectively. Of the 845 genes found altered, only 62 genes were simultaneously altered in all three resistance phenotypes. Using pathway analysis, we found many pathways enriched for each resistance phenotype, but some dominant pathways emerged. The dominant pathways included signaling from the cell surface and cell movement for cisplatin resistance, proteasome regulation and steroid biosynthesis for doxorubicin resistance, and control of translation and oxidative stress for paclitaxel resistance.

**Conclusions:**

Ovarian cancer cells develop drug resistance through different pathways depending on the drug used in the generation of chemoresistance. A better understanding of these mechanisms may lead to the development of novel strategies to circumvent the problem of drug resistance.

## Background

In the United States, ovarian cancer represents 3% of all the new cancer cases in women, but accounts for 5% of all the cancer deaths [1]. This discrepancy is due, in part, to the common resistance of ovarian cancer to current chemotherapy regimens. The vast majority of ovarian cancer patients with advanced disease are treated with surgery followed by adjuvant chemotherapy consisting of a platinum agent (typically carboplatin) in combination with a taxane (paclitaxel). Unfortunately, while most patients initially respond to this combination chemotherapy, a majority of the patients (up to 75%) will eventually relapse within 18 months, many with drug resistant disease [2]. The optimal management of patients with recurrent tumors is unclear, especially for drug resistant disease (by definition, a recurrence that has occurred within 6 months of initial treatment), and various studies have suggested different second line chemotherapy approaches, all with limited success [3]. Ultimately, the frequent development of drug resistance and the lack of alternatives for the treatment of drug resistant disease are responsible for a 5-year survival of approximately 30% in ovarian cancer patients with advanced disease. Indeed, 90% of the deaths from ovarian cancer can be attributed to drug resistance [4].

Studies have shown that ovarian cancer resistance is multifactorial and may involve increased drug inactivation/efflux, increased DNA repair, alterations in cell cycle control, and changes in apoptotic threshold. For example, the copper transporter CTR1 has been shown to mediate cisplatin uptake and cells with decreased CTR1 exhibit increased resistance to cisplatin [5,6]. Another pathway, the PTEN-PI3K-AKT axis, has been suggested to play an important role in the development of drug resistance in several malignancies [7], including ovarian cancer [8-10]. Overall, these studies indicate that a better understanding of the mechanisms of drug action and drug resistance may ultimately lead to new approaches for circumventing resistance and improve patient survival. However, in spite of recent advances, the exact pathways important for the development of drug resistance in ovarian cancer remain unclear. A better understanding of the molecular mechanisms leading to drug resistance may provide new opportunities for the development of strategies for reversing or circumventing drug resistance [4,11].

In this manuscript, we generate novel drug resistant ovarian cancer cell lines independently selected for resistance to cisplatin, doxorubicin or paclitaxel, and we use gene expression profiling to identify genes and pathways that may be important to the development of drug resistance in ovarian cancer.

## Methods

### Cell line and generation of drug resistance sub-lines

The ovarian cancer cell line OV90 was obtained from The American Type Culture Collection (ATCC) and grown in MCDB 105 (Sigma-Aldrich):Media 199 (Invitrogen) containing 15% bovine serum and antibiotics (100 units/ml penicillin and 100 μg/ml streptomycin) at 37°C in a humidified atmosphere of 5% CO_2_. The chemotherapeutic drugs cisplatin, doxorubicin, and paclitaxel were purchased from Sigma. The resistant sub-lines were generated by exposure to the drugs for four to five cycles. For each cycle, the cells were exposed to each individual drug for twenty-four hours, and then transferred to normal media where they were allowed to grow for 2 weeks. Following this two-week period, the cells were re-exposed to the drug to initiate the next cycle.

### Illumina Microarray and data analysis

RNA samples were purified using the RNeasy kit (Qiagen). Biotinylated cRNA was prepared using the Illumina RNA Amplification Kit (Ambion, Inc.) according to the manufacturer's directions starting with approximately 500 ng total RNA. Hybridization to the Sentrix HumanRef-8 Expression BeadChip (Illumina, Inc.), washing and scanning were performed according to the Illumina BeadStation 5006 manual (revision C). Array data processing and analysis was performed using Illumina Bead Studio software. Hierarchical clustering analysis of significant genes was done using an algorithm of the JMP 6.0.0 software. Microarray analysis was performed essentially as described [12]. Raw microarray data were subjected to filtering and z-normalization. Sample quality was assessed using scatterplots and gene sample z-score-based hierarchical clustering. Expression changes for individual genes were considered significant if they met 4 criteria: z-ratio above 1.4 (or below -1.4 for down-regulated genes); false detection rate <0.30; p-value of the pairwise t-test <0.05; and mean background-corrected signal intensity z-score in each comparison group is not negative. This approach provides a good balance between sensitivity and specificity in the identification of differentially expressed genes, avoiding excessive representation of false positive and false negative regulation [13]. All the microarray data are MIAME compliant and the raw data were deposited in Gene Expression Omnibus database [GEO:GSE26465].

### Real-time reverse transcription quantitative-PCR (RT-PCR)

Total RNA was extracted with Trizol (Invitrogen) according to the manufacturer's instructions. RNA was quantified and assessed using the RNA 6000 Nano Kit in the 2100 Bioanalyzer (Agilent Technologies UK Ltd). One μg of total RNA from each cell line was used to generate cDNA using Taqman Reverse Transcription Reagents (PE Applied Biosystems). The SYBR Green I assay and the GeneAmp 7300 Sequence Detection System (PE Applied Biosystems) were used for detecting real-time PCR products. The PCR cycling conditions were as follows: 50°C, 2 min for AmpErase UNG incubation; 95°C, 10 min for AmpliTaq Gold activation; and 40 cycles of melting (95°C, 15 sec) and annealing/extension (60°C for 1 min). PCR reactions for each template were performed in duplicate in 96-well plates. The comparative CT method (PE Applied Biosystems) was used to determine the relative expression in each sample using *GAPDH *as normalization control. The PCR primer sequences are available from the authors.

### Antibodies and Immunoblotting

All the antibodies used for this work were obtained from commercial sources. Anti-ABCB1 was purchased from GeneTex. Anti-SPOCK2 and anti-CCL26 were obtained from R&D Systems. Anti-PRSS8 and anti-MSMB were obtained from Novus Biologicals. Anti-GAPDH was purchased from Abcam. Immunoblotting was performed as previously described [14].

### Pathway Analysis

We used WebGestalt version 2 (http://bioinfo.vanderbilt.edu/webgestalt/) to test for the enrichment of any pathway/terms that may be related to the drug resistance phenotypes. Two different databases (KEGG, and GO) were analyzed using Webgestalt. Overrepresentation analysis was also performed using the Reactome database [15]. Ingenuity Pathway Analysis software (Ingenuity Systems) was used to identify and draw networks relevant to the pathways identified.

### Statistical analysis

Statistical analysis was conducted using Student's *t*-test. A p-value of <0.05 was considered statistically significant.

## Results

### Generation of drug resistant cell lines

**Figure 1:**
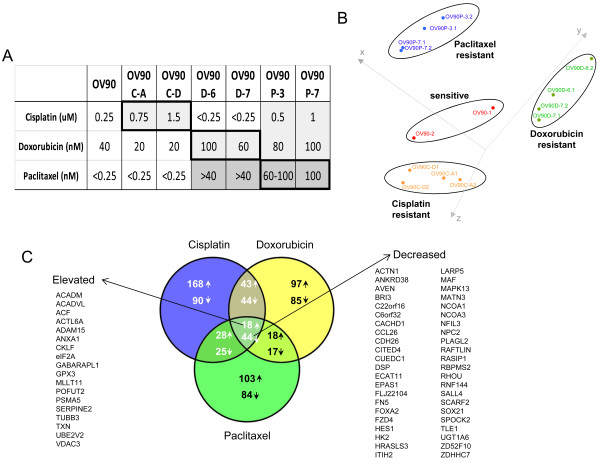
**Establishment of drug resistant cell lines and gene expression profiling**. A. IC_50 _values for the various cell lines used in this study. Thick outlined squares show resistance levels for the drug against which the corresponding cell lines were derived. White squares denote lack of resistance, and light gray squares, moderate resistance. Dark gray indicates drug resistance over 10-fold compared to the parental OV90 line. B. Multi-dimensional scaling plot indicating the cell lines used for the gene expression profiling analysis. Each of the two different resistant clones obtained from the 3 different drugs were cultured and analyzed in duplicate. Two cultures were analyzed for the parental OV90 (OV90-1 and OV90-2). C. Venn diagram representing the number of genes significantly altered in each type of drug resistance. A total of 68 genes were found altered in all three types of resistance generated following exposure to cisplatin, doxorubicin, and paclitaxel.

The drug-sensitive OV90 ovarian cancer cell line was used as a parental line to generate a series of drug resistant cell lines through repeated cycles of drug exposure followed by recovery periods. Using this approach, we generated drug-resistant OV90 sublines through exposure to cisplatin, doxorubicin, or paclitaxel. The lines derived through exposure to cisplatin (OV90C-A, OV90C-D), doxorubicin (OV90D-6, OV90D-7), and paclitaxel (OV90P-3, OV90P-7) all exhibited significant resistance to their corresponding drugs compared to the parental OV90 cell (Figure 1A). When cross resistance was investigated, we found that the cisplatin-derived resistant lines (OV90C-A and OV90C-D) were not cross-resistant to doxorubicin or paclitaxel. In contrast, the doxorubicin-derived resistant cells (OV90D-6 and OV90D-7) exhibited significant cross-resistance to paclitaxel, and the paclitaxel-derived resistant cells (OV90P-3 and OV90P-7) were resistant to both cisplatin and doxorubicin (Figure 1A).

### Microarray analysis of gene expression in drug resistant ovarian cancer cell lines

To identify genes and pathways important in the development of drug resistance, we performed gene expression profiling analysis on the OV90 drug sensitive cell line and on the resistant cell lines using Illumina Sentrix microarrays. For each of the resistance types (cisplatin, doxorubicin, and paclitaxel) two independent sublines were profiled in duplicate (two different cultures). The raw data were deposited in the Gene Expression Omnibus database [GEO:GSE26465]. Multidimensional scaling (MDS) analysis based on gene expression data showed that the cell lines clustered according to the drug used in generating the resistance (Figure 1B), demonstrating that the selection for resistance to different drugs led to overall different patterns of gene expression changes. This suggested different mechanisms of resistance for the different drugs. Comparison of gene expression between sensitive and resistant lines revealed numerous genes differentially expressed. A total of 845 genes (P < 0.05, FDR<0.3) were found altered in at least one drug resistance phenotype (Additional File 1, Figure 1C). Looking at each resistance phenotype individually, 460, 366, and 337 genes were significantly altered (p < 0.01) in the development of resistance to cisplatin, doxorubicin, and paclitaxel, respectively. We identified 18 genes simultaneously elevated in all three drug resistant phenotypes and 44 were downregulated in all three (Figure 1C, Additional File 2). Table 1 shows the top 20 most differentially expressed genes (elevated or decreased) in each one of the three resistance phenotypes. When examining the downregulated genes, only *CCL26 *was found in the top 20 genes in all three resistance phenotypes. None of the top 20 up-regulated genes was found in common between all 3 resistant phenotypes. Interestingly, several genes of the serine protease family (*PRSS *genes) were differentially expressed, although the direction of change was variable (for example, *PRSS2 *was elevated in doxorubicin resistance, but decreased in paclitaxel resistant cells).

**Table 1:** Top 20 genes down- and up-regulated in each drug resistance phenotype

Down-regulated			Up-Regulated		
**Cisplatin**	**Doxorubicin**	**Paclitaxel**	**Cisplatin**	**Doxorubicin**	**Paclitaxel**

CLCA1	APOE	PRSS3	C20orf75	RPIB9	APOA1

CCL26	MSMB	CCL26	WFS1	IL8	GAGE6

RFTN1	CCL26	PRSS2	GNG11	TXNIP	XAGE1

TCN1	ANKRD38	PRSS1	MFGE8	ABCB1	SCRG1

SCARF2	CDH11	RHOU	CEACAM6	PRSS2	GAGE7B

MAPK13	PRSS8	TCN1	MTMR11	PRSS3	ALB

LDHA	APOC1	PRNP	PSG11	GNG11	VSIG1

ECAT11	ITIH2	FKBP11	PAM	CD96	REG4

SPP1	MAF	MSMB	NOS3	LPXN	AFP

DDIT4L	FABP5	LCP1	GAGE6	SGK	FAM112B

APOE	IGSF4	NNMT	CLYBL	MLLT11	RP1-32F7.2

SPOCK2	SOX21	MAF	GAGE7B	CFB	ADH1A

NINJ2	NPC2	ECHDC2	SERPINE2	GADD45A	NMU

THBS1	SCD	ANKRD38	CECR5	MYH4	CTAG2

SOX21	MT1F	WDR72	ADAM15	CXCL6	ADH1C

CD44	RRAGD	CD9	DPYSL3	GABARAPL1	AMBP

RGS4	SPOCK2	MATN2	REG4	POU2F2	MMP1

DDIT4	RENBP	RRAGD	GALR2	PRSS1	PRTFDC1

IGF2	SPINT2	SERPIND1	TFF2	CYR61	GAGE5

GPC3	RFTN1	A2M	EEF1A2	TNFRSF11B	TSPAN12

**Figure 2:**
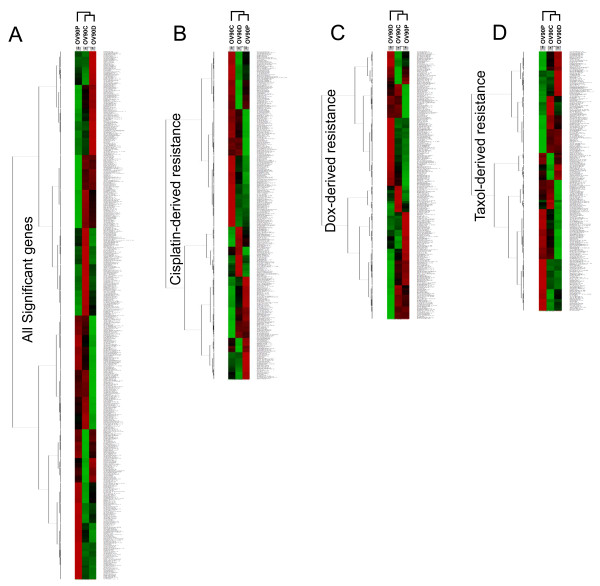
**Genes differentially expressed following the development of drug resistance**. A. Heat map showing the expression of all the significant genes analyzed using the Illumina bead array (845 genes). Changes in gene expression for the 3 pairwise comparisons are included in this analysis (OV90C vs OV90, OV90D vs OV90, and OV90P vs OV90). B. Heat map representing the clustering of genes significantly altered in cisplatin-derived drug resistance. C. Heat map representing the clustering of genes significantly altered in doxorubicin-derived drug resistance. D. Heat map representing the clustering of genes significantly altered in paclitaxel-derived drug resistance.

Hierarchical clustering of the 845 genes significantly altered in at least one condition was performed and is shown in Figure 2A. The variability in the expression patterns among the 3 resistant phenotypes suggested in the Venn diagram (Figure 1C) was evident in the clustering as well (Figure 2A). Clustering was also performed for the genes significantly differentially altered in resistant cell lines developed through cisplatin exposure (Figure 2B), doxorubicin exposure, (Figure 2C) and paclitaxel exposure (Figure 2D). Again, the heat maps showed that the cell lines exhibited little overlap in gene expression changes following the development of resistance to the different drugs.

**Figure 3:**
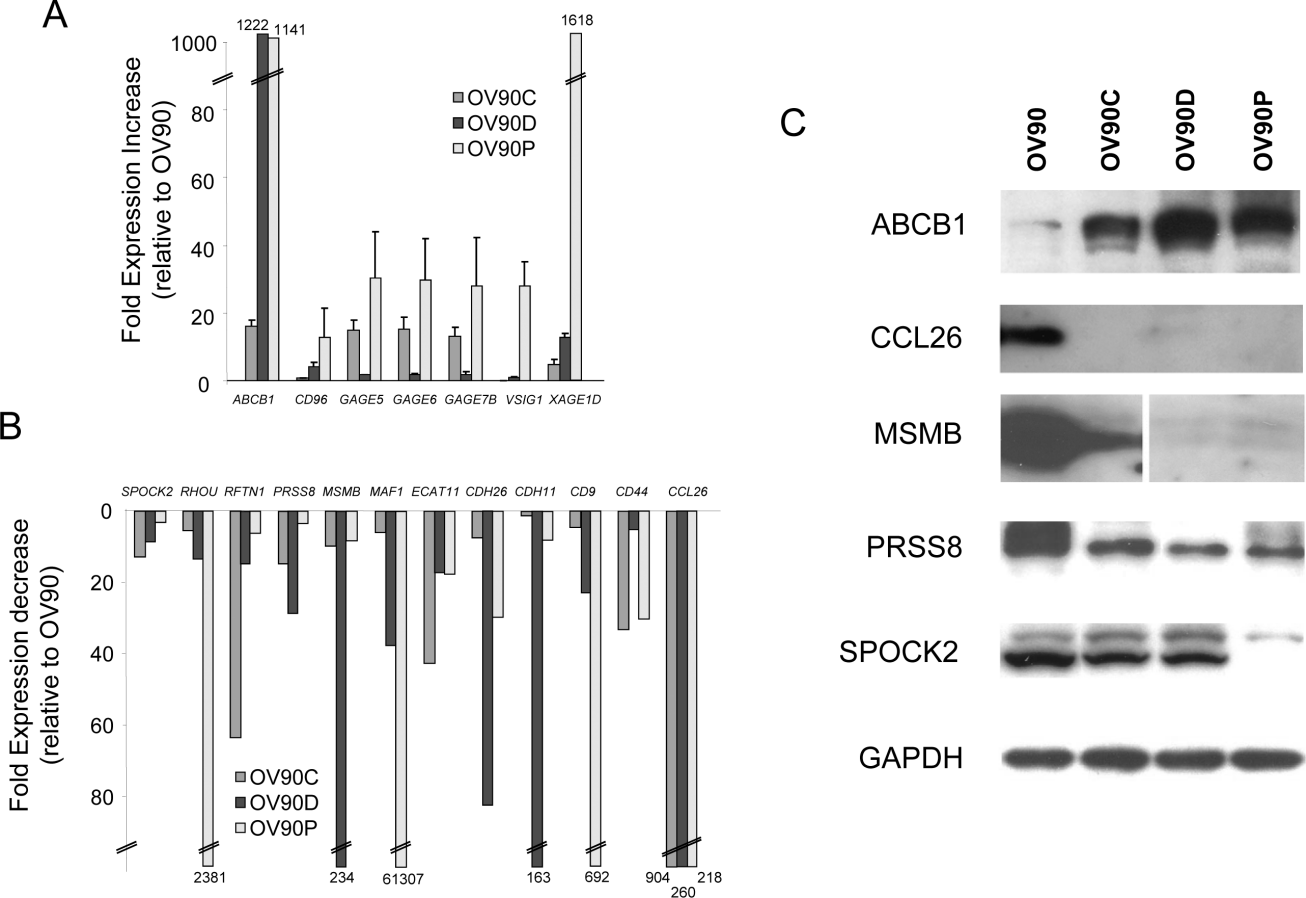
**Validation of selected differentially expressed genes**. A. RT-PCR analysis of genes elevated in drug resistant cells. The y-axis represents fold up-regulation in the different drug resistant cell lines over the parental OV90 cell line. B. RT-PCR analysis of genes decreased in drug resistant cells. The y-axis represents the fold down-regulation of the different resistant cell lines compared to the parental OV90 cell line. C. Immunoblot analysis of selected gene products identified by microarray and RT-PCR as altered in drug resistant cells.

In order to validate the microarray results, we selected a number of highly differentially expressed genes present in Table 1 for validation by RT-PCR. Nineteen genes whose expression patterns were confirmed by RT-PCR are shown in Figure 3A,B. *ABCB1 *was found highly overexpressed, with increases of over 1,000-fold in OV90D and OV90P cells, while the increase in cisplatin-resistant OV90C cells was approximately 15-fold (Figure 3A). Similarly *XAGE1D *expression was also increased 1,000-fold in OV90P cells compare to the OV90 cells. For the other genes analyzed, such as the *GAGE *family genes, *CD96*, and *VSIG1*, the expression levels were increased significantly in various drug resistant cells. In addition, we validated several genes found downregulated in drug resistance (Figure 3B). *CCL26 *was found downregulated more than 200-fold in all three resistant phenotypes compared to drug sensitive cells. *RHOU *and *MAF1 *were decreased over 2,000-fold in OV90-P cells. The other genes analyzed, *SPOCK2*, *RFTN1*, *PRSS8*, *MSMB*, *ECAT11*, *CDH26*, *CDH11*, *CD9*, and *CD44 *were all decreased to various levels in the drug resistant cells.

As further validation, we investigated the protein expression levels of selected candidates by immunoblotting. We found five genes whose protein level changed significantly in the drug resistant cell lines (Figure 3C). Consistent with our RT-PCR findings, the P-glycoprotein (encoded by *ABCB1*), a well-studied protein which has been implicated in multi-drug resistance, was found elevated in all three drug-resistant cell lines, including OV90C, in spite of a relatively small increase in mRNA levels observed in cisplatin cell lines (Figure 3A). On the other hand, the CCL26, PRSS8, and MSMB proteins were found to be significantly decreased in all three drug resistant cell lines. The SPOCK2 protein was only found decreased in the paclitaxel resistant lines (OV90P).

### Pathway analysis of drug resistance

**Figure 4:**
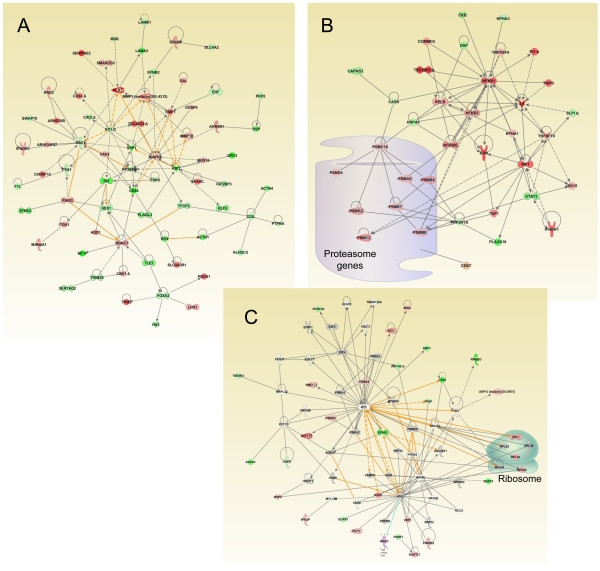
**Network of genes identified using Ingenuity Pathway Analysis**. A. Network including ECM and other genes altered in cisplatin derived resistant cells. B. Network including proteasome genes and other genes altered in doxorubicin resistant cells. C. Network containing translation genes as well as other genes differentially expressed in paclitaxel-derived drug-resistant cells

**Figure 5:**
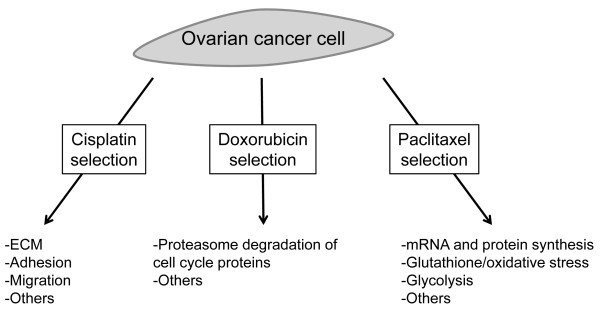
**Model for the development of various resistance phenotypes in ovarian cancer**. Following selection for drug resistance with the indicated drugs, a number of molecular pathways are altered. The molecular pathways identified as altered in the different conditions may be functionally related to the development of drug resistance.

In order to gain some insight into the possible mechanisms important in the development of resistance to these drugs, we performed pathway analysis using the genes that were found significantly differentially expressed in each resistance phenotype. We analyzed the KEGG, GO, and Reactome databases for enrichment of any potential pathways/terms in the 3 different drug resistant cell lines (Table 2). While many pathways were found enriched in each resistance phenotypes, some pathways emerged as consistently identified in the three databases. For example, all the approaches identified various cell surface pathways, including ECM-mediated events as altered in cisplatin resistance. Changes in genes such as *LAMA3*, *LAMA5*, *LAMB1*, *COL17A1*, *CD44*, *ITGA2*, *SDCBP*, and *GPC3 *contributed to these pathways. Ingenuity network analysis was used to identify the relationship between these genes, as well as possible interactions with other genes found altered in our dataset (Figure 4A). In addition, pathways associated with cell movement were also identified in multiple databases as enriched in cisplatin-derived resistant lines. Doxorubicin-derived resistance showed a very strong enrichment for changes in pathways involved proteasome degradation (with changes in proteasome genes *PSMB4*, *PSME2 *, *PSMD8 *, *PSMB7*, *PSME4*, *PSMD14*, *PSMB2*, *PSMC5*, *PSMF1*, *PSMA5*). The p-values for enrichment indicated that this pathway was clearly dominant compared to other pathways (Table 2). Network analysis revealed a vast array of interactions and suggested that many upstream pathways, including NF-κB, may be involved in regulating the proteasome genes identified here (Figure 4B). Paclitaxel resistance exhibited changes in pathways related to mRNA and protein synthesis, and the genes affected included multiple ribosomal genes (*RPS20*, *RPL26*, *RPL10A*, *RPL39*, *RPL7*, and *RPL34*) and translation factors (*EIF4A2*, *EEF1D*). Network analysis shows the possible relationship of the translation pathway with other pathways, including VHL (Figure 4C). Pathways related to oxidative stress (*UGT1A6*, *MAOA*, *GPX3*, and *CYBA*) and glycolysis (*ADH1A*, *HK1*, *ENO3*, *PFKP*, *HK2*, and *ADH1C*) were also found as altered in paclitaxel-derived resistance. Consistent with the fact that gene expression changes were different between the various resistance phenotypes, the dominant pathways were also different (Figure 5), and few pathways were found in common between the various types of resistance (Table 2). When the 62 genes that are found in common between all three resistance phenotypes (Figure 1C) were studied for pathway enrichment, the only pathway found significantly overrepresented was the regulation of fatty acid metabolism and oxidation, which included the differentially-expressed genes *NCOA3*, *NCOA1*, *ACADM*, and *ACADVL*.

**Table 2:** Pathway analysis: Pathways/Terms found enriched in the indicated databases for each of the resistance phenotype are shown.

	KEGG (P < 0.001)	GO (P < 0.1)	Reactome (P < 5e-04)
**Cisp**	Leukocyte transendothelial migration (P = 2.7e-06)	cell-substrate adhesion (adjP = 0.0011)	Nephrin interactions (P = 5.1e-05)
	
	Focal adhesion (P = 4.76e-06)	response to chemical stimulus (adjP = 0.0012)	Recruitment of Proteins To Vesicles (P = 2.7e-04)
	
	ECM-receptor interaction (P = 0.0001)	cellular component movement (adjP = 0.0015)	Activation of PPARA by Fatty Acid (P = 2.8e-04)
	
	Ribosome (P = 0.0001)	homeostasis of number of cells (adjP = 0.0028)	Cell-Cell communication (P = 3.3e-04)
	
	TGF-beta signaling pathway (P = 0.0001)		

**Dox**	Proteasome (P = 2.28e-09)	regulation of ubiquitin-protein ligase	Proteasomal cleavage/Cell cycle (P = 3.2e-06)
	
	Chemokine signaling pathway (P = 7.16e-06)	(mitosis) (adjP = 1.74e-05)	Platelet activation/degranulation (P = 4.7e-06)
	
	Steroid biosynthesis (P = 8.46e-06)		Cholesterol biosynthesis (P = 1.5e-05)
	
	Tight junction (P = 8.91e-06)		
	
	Oocyte meiosis (P = 1.79e-05)		
	
	Leukocyte transendothelial migration (P = 2.1e-05)		

**Tax**	Melanogenesis (P = 4.87e-05)	cellular response to oxidative stress (adjP = 0.08)	Platelet activation/degranulation(P = 7.7e-06)
	
	Glycolysis/Gluconeogenesis (P = 0.0002)	cellular amino acid metabolism (adjP = 0.0782)	Translation (P = 4.2e-04)
	
	Tight junction (P = 0.0002)	hexose metabolic process (adjP = 0.0782)	
	
	Leukocyte transendothelial migration (P = 0.0005)	translation (adjP = 0.0782)	
	
	Glutathione metabolism (P = 0.0005)		
	
	Ribosome (P = 0.0006)		

The p-values for each pathway are indicated.

## Discussion

Drug resistance remains a major obstacle in cancer therapy and significant efforts have been directed at understanding the mechanisms leading to the development of resistance. Gene expression profiling has played a key role in providing us with important clues regarding genes and pathways that may be affected in drug resistance. Overall, the picture that has emerged is that the drug resistance is a multifactorial process involving mechanisms that are both drug- and tissue-dependent. To address these issues in ovarian cancer, we have generated cell lines that are individually resistant to cisplatin, paclitaxel, or doxorubicin. The combination of a platinum compound (cisplatin) and paclitaxel represent the standard initial chemotherapy for ovarian cancer, while doxorubicin has shown some promise in the treatment of recurrent drug-resistant disease [16]. Various studies have investigated drug resistance, but few have compared the drug resistance mechanisms associated with the development of resistance to different drugs.

We found that the gene expression changes associated with the development of drug resistance was dependent on the drug used (Figure 1B), but the individual lines generated from a given drug were extremely similar to each other. This suggests that while cell lines adopted different mechanisms to develop resistance to different drugs, a given drug and conditions seem to favor similar pathways. Interestingly, the patterns of expression associated with cisplatin and doxorubicin resistance were more similar to each other than they were to cell lines developed through paclitaxel exposure (Figure 2A). This is further supported by the observation that the number of differentially expressed genes shared by cisplatin and doxorubicin (149) was greater than the number of genes shared by cisplatin and paclitaxel (115) or paclitaxel and doxorubicin (97) (Figure 1C). Doxorubicin and paclitaxel resistance can both arise through a multi-drug resistance (MDR)-type mechanism, which generally results from overexpression of ATP Binding cassette (ABC) transporters [17], while cisplatin resistance is not believe to have a significant MDR component. On the other hand, cisplatin and doxorubicin are both DNA-damaging agents (albeit acting through different mechanisms), while paclitaxel is a microtubule stabilizing agent. Our data suggest that the overall changes in gene expression tend to reflect the drug target rather than an association with the MDR phenotype.

Overall, relatively few genes were simultaneously altered in the 3 drug resistance phenotypes studied: only 18 genes were elevated and 44 genes decreased. Many of these genes were validated and shown to be differentially expressed at the protein level (Figure 3C). Pathway enrichment analysis of these genes revealed that the most significantly enriched pathway was "fatty acid metabolism and oxidation" (4 genes were part of this pathway). Certain genes consistently downregulated in all the drug resistant lines were particularly interesting. In particular, *MSMB *was found highly downregulated in drug resistant cells at both the mRNA and the protein levels (Figure 3B,C). Interestingly, *MSMB *has been found decreased in prostate cancer and has been suggested to function through its ability to regulate apoptosis [18]. With this function in mind, it is intriguing that we identified *MSMB *as one of the most downregulated genes following the development of drug resistance for all three drugs. These findings suggest that *MSMB *or derivatives may be useful in sensitizing ovarian cancer cells to chemotherapy. In particular, a small peptide derived from the *MSMB *protein has been shown to exhibit anti-tumor properties [19] and has been suggested as a potential therapeutic agent in prostate cancer [20]. It will be interesting to determine whether this peptide may be useful in reversing drug resistance in ovarian cancer and we are currently investigating this enticing possibility. *RFTN1 *is another gene consistently downregulated in all three drug resistance phenotype and it encodes a lipid raft protein. *RFTN1 *is located on chromosome 3p24, a region shown to be frequently deleted in ovarian cancer, including in OV90 cells [21]. This gene has also been shown to be mutated in some ovarian tumors [22], suggesting that it may represent a genuine tumor suppressor gene in this disease. Our results suggest that it may also be involved in drug resistance.

Multiple mechanisms can mediate the development of drug resistance and include 1) changes in the regulation or repair of the primary target of the drug (DNA, microtubule, etc), 2) drug retention (increased influx or decreased uptake), 3) increased drug inactivation or sequestration, 4) signaling pathways that affect survival. For cisplatin, copper transporter CTR1 has been shown to play a crucial role in cisplatin uptake and knockout of the CTR1 alleles can lead to resistance to cisplatin toxicity [5]. On the other hand, paclitaxel and doxorubicin are known substrates for the ATP-dependent efflux pump P-glycoprotein (MDR transporter system, *ABCB1*) and up-regulation of MDR1 has been associated with clinical drug resistance in multiple systems [23]. While we failed to observe changes in the expression of *CTR1 *in cisplatin (or other) resistant lines, we did identify MDR1 (*ABCB1*) as one of our most up-regulated genes in all the resistant phenotypes, including cisplatin resistant cells. Genes of the *GAGE *and *MAGEA *family have also been found elevated in drug resistance. In particular, *MAGEA3*,*6,11,12 *as well as *GAGE2,4,5,6 *and *7 *were found elevated in ovarian cancer cells resistant to paclitaxel and doxorubicin [24]. In this study, we also find *GAGE5,6,7 *and *XAGE1 *to be consistently elevated in the various drug resistant lines, although the levels varied according to the resistance phenotype.

While drug resistance development clearly involves changes in a large number of genes and pathways, we wondered whether pathway analysis may help us identify "dominant" pathways for each drug resistance phenotype. Using pathway analysis, we were indeed able to identify several dominant pathways altered in the different drug resistant cells (Table 2 and Figure 4). Different pathway databases identified different pathways, likely because of variations in annotation and curation, but comparison of the results from different databases allowed us to find pathways that were consistently identified (Figure 4). In cisplatin-derived resistance, we frequently found changes in ECM pathways altered. ECM-Integrin interactions have previously been shown to control cell survival [25] and ECM has been implicated in ovarian cancer drug resistance [26] as well as lung cancer drug resistance [27]. The development of doxorubicin resistance exhibited strong changes in pathways associated with proteasome degradation, This is particularly interesting considering that bortezomib, a proteasome inhibitor, has been found effective in combination therapy with doxorubicin in several studies [28,29]. Because of the specific proteasome genes found altered, as well as the presence of cell cycle genes differentially expressed (such as CDK7), it is likely that the proteasome pathway changes affect the cell cycle. It has been shown that doxorubicin can affect G2/M transition and cyclin B1 activity [30], and changes in the cell cycle may therefore influence the response to doxorubicin through changes in apoptosis sensitivity [31]. Paclitaxel resistance was associated with changes in pathways important for mRNA and protein synthesis, oxidative stress and glycolysis. The exact mechanisms by which these pathways can affect the resistance to paclitaxel remain under investigation, but changes in apoptosis sensitivity is a certain possibility since general mRNA degradation and oxidative stress have been implicated in apoptosis [32,33].

In conclusion, we have generated drug resistant ovarian cancer cell lines through exposure to three different chemotherapeutic drugs and identified gene expression patterns altered during the development of chemoresistance. Among the genes that are consistently elevated we identify previously known genes such as *ABCB1 *and genes of the *MAGEA *family. Among the genes downregulated, we find genes such as *MSMB *and *PRSS *family members that are implicated for the first time in drug resistance. Overall, we find that different drug resistance phenotypes have different expression patterns and we identify many novel genes that may be important in the development of cisplatin, doxorubicin and paclitaxel resistance. Pathway analysis suggests enticing new mechanisms for the development of resistance to cisplatin, doxorubicin, and paclitaxel in ovarian cancer and we find that each resistance phenotype is associated with specific pathway alterations (Figure 5). Whether the identified pathways are causally related to drug resistance remains to be determined and it will be important to follow up these findings with mechanistic studies to better understand the roles of the genes and pathways we have identified.

## Competing interests

The authors declare that they have no competing interests.

## Authors' contributions

CASB generated some of the drug resistant lines, performed the survival experiments on the ovarian cancer cell lines, and helped in drafting the manuscript. KGB participated in the microarray experiments design and analysis. WHW performed the microarray experiments. YZ analyzed the microarray data. PJM conceived the study, oversaw the experiments, analyzed the data, and drafted the manuscript. All the authors in this manuscript have read and approved the final version.

## Supplementary Material

Additional file 1 **Genes differentially expressed between sensitive and resistant cell lines**. The table lists the 845 genes significantly altered in the drug resistant cell lines. The fold change is indicated for each gene in each resistance phenotype (cisplatin, doxorubicin, and paclitaxel). Click here for file

Additional file 2 **Genes simultaneously elevated in all three drug resistant phenotypes**. The table lists all 45 genes simultaneously altered in all three resistance phenotypes (cisplatin, doxorubicin, and paclitaxel), and the fold change is indicated for each. Click here for file
